# Efficacy of various amendments for immobilization of potentially toxic elements in wastewater contaminated soils

**DOI:** 10.1038/s41598-024-65686-x

**Published:** 2024-07-29

**Authors:** Muhammad Zeeshan Manzoor, Ghulam Sarwar, Salman Alamery, Muhammad Ibrahim, Amtul Sami, Bilal Ahmed, Fariha Ahsan, Salma Gul, Kotb A. Attia, Sajid Fiaz, Ikram Ullah

**Affiliations:** 1 Department of Soil and Environmental Sciences, College of Agriculture, University of Sargodha, Sargodha, Pakistan; 2 Department of Biochemistry, College of Science, King Saud University, P.O. Box 2455, 11451 Riyadh, Saudi Arabia; 3 Department of Environmental Sciences, Government College University Faisalabad, Faisalabad, Pakistan; 4 Department of Health Biotechnology, Women University Swabi, Swabi, Pakistan; 5 Department of Structural Engineering, Faculty of Civil Engineering, Doctoral School, Silesian University of Technology, Akademicka 2, 44-100 Gliwice, Poland; 6 Institute of Microbiology, University of Sindh, Jamshoro, 76080 Pakistan; 7 National Centre of Excellence in Physical Chemistry, University of Peshawar, Peshawar, Pakistan; 8 Department of Plant Breeding and Genetics, The University of Haripur, Haripur, 22620 Pakistan; 9 Department of Landscape and Horticulture, Yunnan Agricultural University, Kunming, 650201 China

**Keywords:** Soil, Wastewater, Farm yard manure, Compost, Horse waste, Heavy metals, Environmental sciences, Natural hazards

## Abstract

Farmers are using municipal wastewater either treated or untreated for irrigation because of limited fresh water resources. Wastewater extensively used for irrigation purposes is enriched with many nutrients. The reuse of wastewater is imposing a negative impact on human health and the ecosystem. It is a need of the day to identify and assess issues of the reuse of wastewater. In the current experiment, impact of organic/inorganic amendments was studied to mitigate the toxic effects of pollutants present in wastewater. Soil was brought from the site having consistent use of wastewater and different treatments were applied as per plan. The experiment has 28 treatments with 04 replications. Nine different amendments were used at 3 varying levels. Incubation time of 30 days was given after the addition of all treatments. The results of the study showed the application of FYM @ 5.0% w/w soil reduced soil pH (7.44), EC (2.16 dS m^−1^), SAR (8.14), lead (8.48 mg kg^−1^), cadmium (1.14 mg kg^−1^), nickel (10.55 mg kg^−1^) and arsenic (2.03 mg kg^−1^) when compared with control and other treatments. Usage of compost and horse waste followed FYM. On the basis of this study, it is recommended that wastewater can be used for irrigation purpose after treating with FYM preferably and compost in general.

## Introduction

Gap between supply and demand for water is growing with an abrupt increase in world population. It also poses a threat to human life because it has reached alarming levels in some parts of the globe. Scientific community around the world is working to find new ways to conserve the water and its resources. It is time to refocus attention on a kind of circulating water by reusing municipal wastewater for agricultural purposes particularly irrigation. Water scarcity poses an important task to sustainable farming progress, particularly in the dryland agricultural parts of the world^1^. In a couple of areas, farmers now face the necessity of utilizing urban wastewater for irrigation purposes^2^. As the global population continues to increase and the demand for food rises, water resources dwindle, and the long-distance transport of water imposes substantial strain on agricultural productivity^3^. Utilizing treated urban wastewater for irrigation offers a potential solution by mitigating water shortages, and enhancing water usage efficiency, and it is an economically approachable pathway^4^. The optimal utilization of all available water resources, including treated urban wastewater, becomes crucial in dry and semi-arid climates due to immense pressure on non-renewable water sources, prolonged drought periods, and rapid urbanization^5^. The reuse of treated urban wastewater emerges as an unconventional yet promising water resource that can alleviate water scarcity concerns to some extent^6^. As wastewater contains essential plant nutrients, it becomes imperative to establish proper guidelines for its utilization to minimize the adverse effects associated with wastewater irrigation and maximize its performance^7^. This is indispensable to judge other impacts of wastewater, for example, changes in soil and plant elemental composition, pollutants and heavy metals accumulation^8^.

Elements with metallic characteristics including malleability, ductility, conductivity, cation stability, and ligand selectivity are known as heavy metals. These often have atomic weights above 20 and large densities^9^. Heavy metals, also known as metalloids, such as Pb, Cd, Hg, and As are regarded as serious hazards because these have no beneficial effects on organisms and are extremely dangerous to both plants and animals. Heavy metal-contaminated soil frequently has poor biological, physical, and chemical characteristics. Hence, soil contamination has been recognized progressively as a global environmental problem^10,10^.

Organic soil additives play important roles in enhancing the structure and texture of soil and also increase the organic content in the soil including retention capacity for water and nutrients. Application of biochar, animal manure and compost are some common organic practices or soil amendments to minimize the impact of potentially toxic elements (PTSs)^12^. Rani and Singh^13^ focused on addressing heavy metal contamination in the soil through the addition of various materials of organic nature like farm and poultry manure, compost and biochar. Their findings demonstrated the effectiveness of these amendments in reducing heavy metal concentrations and enhancing soil quality. Similarly, Rashmi et al.^14^ emphasized the eco-friendly approach of using soil amendments to promote soil health and sustainable oilseed production. Their research explored various organic amendments and their potential to recover soil fertility as well as crop production. Efficacy of organic amendments in reducing the phytotoxicity of cadmium (Cd) assimilation in wheat was explored and it was noted that organic amendments can reduce cadmium toxicity and plant uptake^15^. Likewise, impact of organic fertilizers on the quantity and quality of soil organic matter in conventional agriculture was studied and the positive outcome of organic fertilizers in increasing soil organic matter and improving the overall condition of the soil was noted^16^.

Keeping in view the issues related with the use of wastewater in untreated form, various amendments were utilized to overcome problems of heavy metal toxicity associated with the use of wastewater irrigation. Hence, various amendments were used to ameliorate wastewater being utilized for irrigation purposes in semi-arid climates. This study investigates the potential of organic fertilizers to enhance soil fertility and promote sustainable crop productivity in heavy metal-contaminated soils, building on previous research that has demonstrated the effectiveness of chemical additives and organic amendments in mitigating soil pollution and improving crop growth and antioxidant activity. In this study, impact of various inorganic and organic amendments was studied to mitigate the toxic effects of pollutants present in wastewater when used as a source of irrigation. Keeping in view the current scenario of water deficiency, current research was designed given the following hypothesis;

Inorganic and organic amendments can immobilize heavy metals from polluted soil.

To achieve this hypothesis, the experiment was planned with the following objectives:

1. To appraise the efficiency of organic and inorganic materials to mitigate wastewater-created soil pollutant toxicity and shortlisting of the most effective measures
2. To frame recommendations for farmers to use wastewater irrigation after managing deleterious effects to avoid probable pollution.

## Materials and methods

### Collection of samples

In this experiment, efficiency of different inorganic and organic amendments was evaluated to mitigate the toxicity of various pollutants in the soil samples collected from farmer’s field located at Raza Garden, Sillanwali Road, Sargodha and conclude final recommendations for farmers about the use of wastewater irrigation. Soil samples were collected from the depth of 0–15 cm using standard sampling methods.

### Layout of experiment

A detailed description of all treatment is shown as follows:

**Nature of study** Laboratory experiment.

**Treatments** 28 with 04 replications.

T_1_ = Control (polluted soil), T_2_ = Soil + FYM @ 1% w/w soil, T_3_ = Soil + FYM @ 2.5% w/w soil, T_4_ = Soil + FYM @ 5.0% w/w soil, T_5_ = Soil + compost @ 1% w/w soil, T_6_ = Soil + compost @ 2.5% w/w soil, T_7_ = Soil + compost @ 5.0% w/w soil, T_8_ = Soil + poultry manure @ 1% by weight of soil, T_9_ = Soil + poultry manure @ 2.5% w/w soil, T_10_ = Soil + poultry manure @ 5.0% w/w soil, T_11_ = Soil + press mud @ 1% w/w soil, T_12_ = Soil + press mud @ 2.5% w/w soil, T_13_ = Soil + press mud @ 5.0% w/w soil, T_14_ = Soil + biochar @ 1% w/w soil, T_15_ = Soil + biochar @ 2.5% w/w soil, T_16_ = Soil + biochar @ 5.0% w/w soil, T_17_ = Soil + rice husk @ 1% w/w soil, T_18_ = Soil + rice husk @ 2.5% w/w soil, T_19_ = Soil + rise husk @ 5.0% w/w soil, T_20_ = Soil + wheat straw @ 1% w/w soil, T_21_ = Soil + wheat straw @ 2.5% w/w soil, T_22_ = Soil + wheat straw @ 5% w/w soil, T_23_ = Soil + gypsum @ 1.0% w/w soil, T_24_ = Soil + gypsum @ 2.5% w/w soil, T_25_ = Soil + gypsum @ 5.0% w/w soil, T_26_ = Soil + horse waste @ 1% w/w soil, T_27_ = Soil + horse waste @ 2.5% w/w soil and T_28_ = Soil + horse waste @ 5.0% w/w soil.

After 30 days’ incubation, all soil samples were analyzed, and values of heavy metals were measured.

### Measurements of soil samples

All Laboratory analysis for soil, wastewater and organic amendment’s samples was made according to the methods written in Hand Book 60 of U.S Laboratory Staff^17^ or otherwise mentioned. Soil texture, EC, pH, SAR, heavy metals (lead, cadmium, nickel, and arsenic), and organic matter content were analyzed to identify the characterization of wastewater-polluted soil samples (Table 1).

**Table 1:** Characteristics of soil used during experimentation.

Sr. No.	Characteristics	Unit	Value
1	EC	dS m^−1^	2.75
2	pH	–	8.26
3	SAR	–	13.15
4	Organic matter	%	0.69
5	Lead	mg kg^−1^	12.17
6	Cadmium	mg kg^−1^	1.67
7	Nickel	mg kg^−1^	13.52
8	Arsenic	mg kg^−1^	4.17
9	Sand	%	38
10	Silt	%	30
11	Clay	%	32
12	Textural Class	–	Clay Loam

### Application of amendments

In laboratory study different amendments such as FYM, compost, poultry manure, press mud, biochar, wheat straw, rice husk, horse waste and gypsum were tested (Table 2) and evaluated for their efficiency when applied to polluted soil at various rates.

**Table 2:** Chemical composition of amendments used in experiment.

Sr. No.	Determinations	Unit	FYM	Compost	Poultry Manure	Press Mud	Biochar	Rice Husk	Wheat Straw	Gypsum	Horse Waste
1	pH (1:1)	–	7.92	7.29	5.70	7.8	7.1	7.1	8.2	6.7	8.23
2	EC	dS m^−1^	12.78	6.24	6.84	2.2	0.75	1.06	1.75	2.1	6.92
3	Organic Matter	%	35.98	34.89	26.54	21.0	21.14	19.34	19.25	–	35.62
4	Organic Carbon	%	20.92	20.28	15.43	12.21	12.29	11.24	11.19	–	20.17
5	Total nitrogen	%	1.81	1.72	1.35	1.09	1.11	0.95	0.97	–	1.75
6	C/N ratio	–	11.56	11.79	11.43	11.20	11.07	11.83	11.54	–	11.53
7	Lead	mg kg^−1^	0.54	0.58	1.29	0.05	0.65	0.27	–	3.52	0.97
8	Cadmium	mg kg^−1^	0.38	0.32	0.41	0.04	0.13	0.19	–	0.95	0.17
9	Nickel	mg kg^−1^	1.02	1.08	1.63	0.03	0.73	0.41	0.15	0.58	1.00
10	Arsenic	mg kg^−1^	–	–	1.67	–	0.24	–	–	0.22	–

### Measurements of wastewater samples

Wastewater used in this study was analyzed for EC, pH, SAR, RSC, heavy metals like lead, cadmium, nickel, and arsenic using standard analytical methods as quoted in Hand Book 60 of U.S Laboratory Staff^17^ or otherwise mentioned (Table 3).

**Table 3:** Characteristics of wastewater used during laboratory experimentation.

Sr. No.	Characteristics	Unit	Value
1	EC	dS m^−1^	3.65
2	pH	–	7.07
3	SAR	–	7.12
4	RSC	me L^−1^	1.19
5	Pb	mg L^−1^	2.60
6	Cd	mg L^−1^	0.01
7	Ni	mg L^−1^	0.14
8	As	mg L^−1^	0.08

### Soil texture

The Hydrometer method was used to determine soil textural class. The International Textural Triangle, which offers a categorization system based on the proportions of sand, silt, and clay in the soil^18^, was used to identify the soil textural class.

### Residual sodium carbonates (RSC) measurement (meL^-1^)

Equation was used to compute the residual sodium carbonate (RSC):

$${\text{RSC}}\, = \,\left( {{\text{CO}}_{{3}}^{{{-}{2}}} \, + \,{\text{HCO}}_{{3}}^{ - } } \right) - \left( {{\text{Ca}}^{ + + } \, + \,{\text{Mg}}^{ + + } } \right).$$

All appeared as me L^−1^.

### Soil organic matter measurement (%)

The method of Walkley and Black^19^ was used to calculate the total soil organic carbon content.

### Heavy metals measurements (Pb, Cd, Ni and As)

After the digestion of soil and plant samples, heavy metals (Pb, Cd, Ni, & As) were determined using an atomic absorption spectrophotometer^1^.

### Statistical analysis

Data collected were subjected to statistical analysis using software program “Statistics 8.1,”. The statistical analysis used the Fisher’s analysis of variance (ANOVA) method. According to the procedure outlined by Steel et al.^20^, significant differences between treatment effects were identified using the LSD test at a 1% probability level (Table 4).

**Table 4:** Statistical analysis of various studied parameters.

SOV	DF	Soil pH	Soil EC	Soil SAR	OM	Pb	Cd	Ni	AS
Treatment	27	0.243**	0.123**	7.060**	0.004**	5.705**	0.073**	3.079**	1.980**
Error	56	0.001	0.001	0.001	0.001	0.001	0.001	0.001	0.001

*SOV* Source of variance, *DF* Degree of freedom, **Significant at *P* < 0.01.

### Ethical approval

Experimental research and field studies on plants (either cultivated or wild), including the collection of plant material, complied with relevant institutional, national, and international guidelines and legislation. A prior approval was undertaken from Offices of Research, Innovation and Commercialization, University of Sargodha, Pakistan.

## Results

Current study was carried out at College of Agriculture, University of Sargodha, Sargodha, Punjab, Pakistan. In this study, impact of various amendments was evaluated to mitigate the toxic effects of pollutants present in wastewater when used as the source of irrigation.

### Impact of organic amendments on soil pH

Soil pH is the single soil characteristic, which elucidates an overall picture of the medium for plant growth including nutrient supply trend, fate of added nutrients, salinity/sodicity status and soil aeration, soil mineralogy and ultimate weather conditions of the region. Hence, a decrease in soil pH due to any land management strategy is always appreciable and result in ultimate conversion of soil medium towards favorable one. Soil pH was significantly affected by all applied amendments when compared with control (Table 5). The experimental results revealed that all organic amendments significantly reduced soil pH levels compared to the control treatment (T1), which had the highest pH value of 8.26. The treatment with the highest concentration of farmyard manure (FYM) (T4) achieved the lowest pH value of 7.44. The addition of compost at varying rates (T5–T7) resulted in pH values ranging from 7.57 to 7.45. Similarly, the application of poultry manure at different rates (T8–T10) yielded pH values between 8.13 and 7.94, with a statistically significant difference observed between T9 and T10. Moreover, the addition of horse waste at various rates (T26–T28) resulted in pH values ranging from 7.65 to 7.45. Notably, all organic amendments applied at a rate of 5% by weight outperformed the 1.0 and 2.5% application rates in reducing soil pH levels.

**Table 5:** Impact of various treatments on soil pH.

Sr. No.	Treatments	pH	Sr. No.	Treatments	pH
T_1_	Control	8.26^a^	T_15_	Soil + biochar @ 2.5% W/W Soil	7.94^e^
T_2_	Soil + FYM @ 1% W/W Soil	7.56^f,g^	T_16_	Soil + biochar @ 5.0% W/W Soil	7.92^e^
T_3_	Soil + FYM @ 2.5% W/W Soil	7.45^h,i^	T_17_	Soil + ricehusk @ 1% W/W Soil	8.16^b,c^
T_4_	Soil + FYM @ 5.0% W/W Soil	7.44^i^	T_18_	Soil + ricehusk @ 2.5% W/W Soil	8.12^b,c,d^
T_5_	Soil + compost @ 1% W/W Soil	7.57^f,g^	T_19_	Soil + ricehusk @ 5.0% W/W Soil	8.09^c,d^
T_6_	Soil + compost @ 2.5% W/W Soil	7.51^g,h,i^	T_20_	Soil + wheat straw @ 1% W/W Soil	8.19^a,b^
T_7_	Soil + compost @ 5.0% W/W Soil	7.45^h,i^	T_21_	Soil + wheat straw @ 2.5% W/W Soil	8.16^b,c^
T_8_	Soil + poultry manure @ 1% W/W Soil	8.13^b,c,d^	T_22_	Soil + wheat straw @ 5.0% W/W Soil	8.14^b,c,d^
T_9_	Soil + poultry manure @ 2.5% W/W Soil	8.07^d^	T_23_	Soil + gypsum @ 1% W/W Soil	8.15^b,c,d^
T_10_	Soil + poultry manure @ 5.0% W/W Soil	7.94^e^	T_24_	Soil + gypsum @ 2.5% W/W Soil	8.13^b,c,d^
T_11_	Soil + pressmud @ 1% W/W Soil	8.17^a,b,c^	T_25_	Soil + gypsum @ 5.0% W/W Soil	8.11^b,c,d^
T_12_	Soil + pressmud @ 2.5% W/W Soil	7.96^e^	T_26_	Soil + horse waste @ 1% W/W Soil	7.65^f^
T_13_	Soil + pressmud @ 5.0% W/W Soil	7.95^e^	T_27_	Soil + horse waste @ 2.5% W/W Soil	7.54^g,h^
T_14_	Soil + biochar @ 1% W/W Soil	7.96^e^	T_28_	Soil + horse waste @ 5.0% W/W Soil	7.45^h,i^
Tukey HSD		0.0929			

*FYM* Farm yard manure, *W/W* weight by weight.

The alphabets e.g., a, b, c represent the statistical grouping of treatments based on their pH values.

### Impact of organic amendments on soil EC

Electrical conductivity is a soil parameter that indicates indirectly the total concentration of soluble salts and is a direct measurement of salinity. Salinity is a problem of arid and semi-arid regions in the world. The critical limit of this soil character is 4.0 dS.m^−1^ above which plants face problems of water uptake due to physiological unavailability, osmotic effects due to decrease of water potential and restricted nutrient uptake that may be due to specific ion effect. Data indicated a general decreasing trend in electrical conductivity of soil when organic amendments were applied to the soil (Table 6). Statistical analysis revealed significant differences among the various treatments. The control treatment (T1) had the highest electrical conductivity (EC) value of 2.74 dS m^−1^, while the other treatments ranged from 2.16 to 2.72 dS m^−1^. Notably, the organic amendments tested, including farmyard manure (FYM) (T2–T4), compost (T5–T7), biochar (T14–T16), rice husk (T17–T19), and wheat straw (T20–T22), resulted in lower EC values compared to the control. In contrast, treatments with poultry manure (T8–T10) and horse waste (T26–T28) had slightly higher EC values than the other amendments. Specifically, the compost treatments (T5–T7) at different rates (1.0, 2.5, or 5% by weight) yielded EC values of 2.26, 2.21, and 2.16 dS m^−1^, respectively. Similarly, the poultry manure treatments (T8–T10) at different rates resulted in EC values of 2.69, 2.65, and 2.62 dS m^−1^, respectively.

**Table 6:** Impact of various treatments on soil EC.

Sr. No.	Treatments	EC	Sr. No.	Treatments	EC
T_1_	Control	2.74^a^	T_15_	Soil + biochar @ 2.5% w/w Soil	2.65^c,d,e,f^
T_2_	Soil + FYM @ 1% w/w Soil	2.20^j^	T_16_	Soil + biochar @ 5.0% w/w Soil	2.64^d,e,f^
T_3_	Soil + FYM @ 2.5% w/w Soil	2.17^j^	T_17_	Soil + rice husk @ 1% w/w Soil	2.72^a,b^
T_4_	Soil + FYM @ 5.0% w/w Soil	2.16^j^	T_18_	Soil + rice husk @ 2.5% w/w Soil	2.69^b,c^
T_5_	Soil + compost @ 1% w/w Soil	2.26^i^	T_19_	Soil + rice husk @ 5.0% w/w Soil	2.66^c,d,e,f^
T_6_	Soil + compost @ 2.5% w/w Soil	2.21^j^	T_20_	Soil + wheat straw @ 1% w/w Soil	2.69^b,c^
T_7_	Soil + compost @ 5.0% w/w Soil	2.16^j^	T_21_	Soil + wheat straw @ 2.5% w/w Soil	2.68^b,c,d^
T_8_	Soil + poultry manure @ 1% w/w Soil	2.69^b,c^	T_22_	Soil + wheat straw @ 5.0% w/w Soil	2.66^c,d,e,f^
T_9_	Soil + poultry manure @ 2.5% w/w Soil	2.65^c,d,e,f^	T_23_	Soil + gypsum @ 1% w/w Soil	2.69^b,c^
T_10_	Soil + poultry manure @ 5.0% w/w Soil	2.62^e,f^	T_24_	Soil + gypsum @ 2.5% w/w Soil	2.67^b,c,d,e^
T_11_	Soil + pressmud @ 1% w/w Soil	2.66^c,d,e,f^	T_25_	Soil + gypsum @ 5.0% w/w Soil	2.65^c,d,e,f^
T_12_	Soil + pressmud @ 2.5% w/w Soil	2.64^d,e,f^	T_26_	Soil + horse waste @ 1% w/w Soil	2.45^g^
T_13_	Soil + pressmud @ 5.0% w/w Soil	2.62^f^	T_27_	Soil + horse waste @ 2.5% w/w Soil	2.42^g^
T_14_	Soil + biochar @ 1% w/w Soil	2.67^c,d,e^	T_28_	Soil + horse waste @ 5.0% w/w Soil	2.37^h^
Tukey HSD	0.0486				

*FYM* Farm yard manure, *W/W* weight by weight.

The alphabets e.g., a, b, c represent the statistical grouping of treatments based on their EC values.

### Impact of organic amendments on soil SAR

Sodium adsorption ratio (SAR) is yardstick used to measure the sodicity of a soil. Sodicity is the accumulation of sodium ion in excessive quantities, which hinder plant growth directly or through the impairment of physical soil conditions. Data indicated that all treatments have statistically significant impact on SAR of the soil (Table 7). Statistical analysis revealed significant differences in Sodium Adsorption Ratio (SAR) values among the various treatments. The control treatment (T1) had the highest SAR value of 13.14, while treatment T4 had the lowest value of 8.14. Treatments T6 (soil + compost at 2.5% w/w soil) and T7 (soil + compost at 5.0% w/w soil) also showed relatively low SAR values of 8.25 and 8.21, respectively. The use of horse waste was found to be second only to compost in reducing soil SAR. However, the application of amendments such as poultry manure (T8–T10), pressmud (T11–T13), biochar (T14–T16), rice husk (T17–T19), wheat straw (T20–T22), gypsum (T23–T25), and horse waste (T26–T28) was less effective in reducing SAR values compared to farmyard manure (FYM), compost, and horse waste.

**Table 7:** Impact of various treatments on SAR of soil.

Sr. No.	Treatments	SAR	Sr. No.	Treatments	SAR
T_1_	Control	13.14^a^	T_15_	Soil + biochar @ 2.5% W/W Soil	9.27^m^
T_2_	Soil + FYM @ 1% W/W Soil	8.32^n,o^	T_16_	Soil + biochar @ 5.0% W/W Soil	9.23^m^
T_3_	Soil + FYM @ 2.5% W/W Soil	8.25^o,p,q^	T_17_	Soil + ricehusk @ 1% W/W Soil	11.56^d,e^
T_4_	Soil + FYM @ 5.0% W/W Soil	8.14^r^	T_18_	Soil + ricehusk @ 2.5% W/W Soil	11.52^e^
T_5_	Soil + compost @ 1% W/W Soil	8.32^n,o^	T_19_	Soil + ricehusk @ 5.0% W/W Soil	11.45^f^
T_6_	Soil + compost @ 2.5% W/W Soil	8.25^p,q^	T_20_	Soil + wheat straw @ 1% W/W Soil	11.69^b^
T_7_	Soil + compost @ 5.0% W/W Soil	8.21^q^	T_21_	Soil + wheat straw @ 2.5% W/W Soil	11.66^b^
T_8_	Soil + poultry manure @ 1% W/W Soil	10.86^g^	T_22_	Soil + wheat straw @ 5.0% W/W Soil	11.63^b,c^
T_9_	Soil + poultry manure @ 2.5% W/W Soil	10.74^h^	T_23_	Soil + gypsum @ 1% W/W Soil	11.65^b,c^
T_10_	Soil + poultry manure @ 5.0% W/W Soil	10.66^i,j^	T_24_	Soil + gypsum @ 2.5% W/W Soil	11.63^b,c^
T_11_	Soil + pressmud @ 1% W/W Soil	10.68^h,i^	T_25_	Soil + gypsum @ 5.0% W/W Soil	11.59^c,d^
T_12_	Soil + pressmud @ 2.5% W/W Soil	10.60^j,k^	T_26_	Soil + horse waste @ 1% W/W Soil	8.36^n^
T_13_	Soil + pressmud @ 5.0% W/W Soil	10.57^k^	T_27_	Soil + horse waste @ 2.5% W/W Soil	8.33^n^
T_14_	Soil + biochar @ 1% W/W Soil	9.34^l^	T_28_	Soil + horse waste @ 5.0% W/W Soil	8.31^n,o,p^
Tukey HSD	0.067				

*FYM* Farm yard manure, *W/W* weight by weight.

The alphabets e.g., a, b, c represent the statistical grouping of treatments based on their SAR values.

### Impact of organic amendments on soil organic matter

Organic matter is regarded as the ultimate source of nutrients and microbial activity in the soil. It is the deciding factor in soil structure, water holding capacity, infiltration rate, aeration and porosity of the soil. Thus, if only one soil parameter of productivity is to be considered that may be organic matter. Usage of different organic amendments enhanced the content of soil organic matter significantly when compared with control (Table 8). The organic matter content, which was at its lowest level of 0.69% in the control treatment (T1), was significantly enhanced to a highest value of 0.82% in treatment T4 (FYM @ 5% by weight). The impact of compost, horse waste, and poultry manure was found to be comparable to that of FYM, with a positive effect on soil organic matter content. However, the use of press mud and gypsum was relatively less effective in increasing soil organic matter levels compared to other amendments.

**Table 8:** Impact of various treatments on OM of soil.

Sr. No.	Treatments	OM	Sr. No.	Treatments	OM
T_1_	Control	0.69^e,f,g,h^	T_15_	Soil + biochar @ 2.5% W/W Soil	0.70^d,e,f,g,h^
T_2_	Soil + FYM @ 1% W/W Soil	0.77^b,c^	T_16_	Soil + biochar @ 5.0% W/W Soil	0.72^d,e,f,g,h^
T_3_	Soil + FYM @ 2.5% W/W Soil	0.80^a,b^	T_17_	Soil + ricehusk @ 1% W/W Soil	0.69^e,f,g,h^
T_4_	Soil + FYM @ 5.0% W/W Soil	0.82^a^	T_18_	Soil + ricehusk @ 2.5% W/W Soil	0.70^d,e,f,g,h^
T_5_	Soil + compost @ 1% W/W Soil	0.73^c,d,e^	T_19_	Soil + ricehusk @ 5.0% W/W Soil	0.72^d,e,f,g,h^
T_6_	Soil + compost @ 2.5% W/W Soil	0.75^c,d^	T_20_	Soil + wheat straw @ 1% W/W Soil	0.70^e,f,g,h^
T_7_	Soil + compost @ 5.0% W/W Soil	0.80^a,b^	T_21_	Soil + wheat straw @ 2.5% W/W Soil	0.72^d,e,f,g,h^
T_8_	Soil + poultry manure @ 1% W/W Soil	0.69^e,f,g,h^	T_22_	Soil + wheat straw @ 5.0% W/W Soil	0.72^d,e,f^
T_9_	Soil + poultry manure @ 2.5% W/W Soil	0.71^d,e,f,g,h^	T_23_	Soil + gypsum @ 1% W/W Soil	0.68^g,h^
T_10_	Soil + poultry manure @ 5.0% W/W Soil	0.72^d,e,f,g^	T_24_	Soil + gypsum @ 2.5% W/W Soil	0.69^e,f,g,h^
T_11_	Soil + pressmud @ 1% W/W Soil	0.68^g,h^	T_25_	Soil + gypsum @ 5.0% W/W Soil	0.71^d,e,f,g,h^
T_12_	Soil + pressmud @ 2.5% W/W Soil	0.68^f,g,h^	T_26_	Soil + horse waste @ 1% W/W Soil	0.70^d,e,f,g,h^
T_13_	Soil + pressmud @ 5.0% W/W Soil	0.70^d,e,f,g,h^	T_27_	Soil + horse waste @ 2.5% W/W Soil	0.73^c,d,e,f^
T_14_	Soil + biochar @ 1% W/W Soil	0.67^h^	T_28_	Soil + horse waste @ 5.0% W/W Soil	0.75^c,d^
Tukey HSD	0.045				

*FYM* Farm yard manure, *W/W* weight by weight.

The alphabets e.g., a, b, c represent the statistical grouping of treatments based on their OM values.

### Impact of organic amendments on Pb in soil

The data revealed a consistent decline in soil lead (Pb) concentrations with the application of organic amendments (Table 9). Statistical analysis confirmed significant differences among treatments. The control treatment (T1) had the highest Pb concentration of 12.16 mg kg^−1^, while the other treatments ranged from 11.96 to 8.48 mg kg^−1^. Among the tested organic amendments, treatments with farmyard manure (FYM) (T2–T4), compost (T5–T7), and horse waste (T26–T28) were the most effective in reducing Pb concentrations compared to the control. Treatments with poultry manure (T8–T10), press mud (T11–T13), rice husk (T17–T19), wheat straw (T20–T22), and gypsum (T23–T25) were less effective in reducing Pb levels, but still showed a decrease in Pb concentration compared to the control, indicating their potential for soil remediation.

**Table 9:** Impact of various treatments on Pb in soil.

Sr. No.	Treatments	Pb	Sr. No.	Treatments	Pb
T_1_	Control	12.16^a^	T_15_	Soil + biochar @ 2.5% W/W Soil	10.85^k^
T_2_	Soil + FYM @ 1% W/W Soil	8.65^p^	T_16_	Soil + biochar @ 5.0% W/W Soil	10.74^l^
T_3_	Soil + FYM @ 2.5% W/W Soil	8.56^q^	T_17_	Soil + ricehusk @ 1% W/W Soil	11.65^f,g^
T_4_	Soil + FYM @ 5.0% W/W Soil	8.48^q^	T_18_	Soil + ricehusk @ 2.5% W/W Soil	11.60^g,h^
T_5_	Soil + compost @ 1% W/W Soil	8.95^m^	T_19_	Soil + ricehusk @ 5.0% W/W Soil	11.64^f,g^
T_6_	Soil + compost @ 2.5% W/W Soil	8.90^m,n^	T_20_	Soil + wheat straw @ 1% W/W Soil	11.74^d,e^
T_7_	Soil + compost @ 5.0% W/W Soil	8.75^o^	T_21_	Soil + wheat straw @ 2.5% W/W Soil	11.56^h^
T_8_	Soil + poultry manure @ 1% W/W Soil	11.96^b^	T_22_	Soil + wheat straw @ 5.0% W/W Soil	11.48^i^
T_9_	Soil + poultry manure @ 2.5% W/W Soil	11.88^b,c^	T_23_	Soil + gypsum @ 1% W/W Soil	11.75^de^
T_10_	Soil + poultry manure @ 5.0% W/W Soil	11.81^c,d^	T_24_	Soil + gypsum @ 2.5% W/W Soil	11.71^ef^
T_11_	Soil + pressmud @ 1% W/W Soil	11.81^c,d^	T_25_	Soil + gypsum @ 5.0% W/W Soil	11.64^f,g,h^
T_12_	Soil + pressmud @ 2.5% W/W Soil	11.73^d,e^	T_26_	Soil + horse waste @ 1% W/W Soil	8.92^m,n^
T_13_	Soil + pressmud @ 5.0% W/W Soil	11.65^f,g^	T_27_	Soil + horse waste @ 2.5% W/W Soil	8.89^m,n^
T_14_	Soil + biochar @ 1% W/W Soil	10.95^j^	T_28_	Soil + horse waste @ 5.0% W/W Soil	8.85^n^
Tukey HSD	0.078				

*FYM* Farm yard manure, *W/W* weight by weight.

The alphabets e.g., a, b, c represent the statistical grouping of treatments based on their Pb values.

### Impact of organic amendments on Cd in soil

The data revealed that all treatments had a statistically significant impact on soil cadmium concentrations (Table 10). Statistical analysis confirmed significant differences in cadmium concentrations among the various treatments. The control treatment (T1) had the highest cadmium concentration of 1.66 mg kg^−1^, while treatment T4 had the lowest concentration of 1.14 mg kg^−1^. Treatments T6 (soil + compost at 2.5% w/w soil) and T7 (soil + compost at 5.0% w/w soil) also showed relatively low cadmium concentrations of 1.17 and 1.16 mg kg^−1^, respectively. The use of horse waste was found to be second only to compost in reducing soil cadmium concentrations. However, the application of amendments such as poultry manure (T8–T10), press mud (T11–T13), biochar (T14–T16), rice husk (T17–T19), wheat straw (T20–T22), and gypsum (T23–T25) was less effective in reducing cadmium levels compared to farmyard manure (FYM), compost, and horse waste. Nevertheless, the cadmium concentrations in these treatments were lower than in the control, indicating their potential for soil remediation.

**Table 10:** Impact of various treatments on Cd of soil.

Sr. No.	Treatments	Cd	Sr. No.	Treatments	Cd
T_1_	Control	1.66^a^	T_15_	Soil + biochar @ 2.5% W/W Soil	1.43^h^
T_2_	Soil + FYM @ 1% W/W Soil	1.20^j,k^	T_16_	Soil + biochar @ 5.0% W/W Soil	1.42^h^
T_3_	Soil + FYM @ 2.5% W/W Soil	1.17^k,l^	T_17_	Soil + rice husk @ 1% W/W Soil	1.56^b,c^
T_4_	Soil + FYM @ 5.0% W/W Soil	1.14^l^	T_18_	Soil + rice husk @ 2.5% W/W Soil	1.54^b,c,d,e,f^
T_5_	Soil + compost @ 1% W/W Soil	1.23^j^	T_19_	Soil + rice husk @ 5.0% W/W Soil	1.51^e,f^
T_6_	Soil + compost @ 2.5% W/W Soil	1.17^k,l^	T_20_	Soil + wheat straw @ 1% W/W Soil	1.54^b,c,d,e^
T_7_	Soil + compost @ 5.0% W/W Soil	1.16^k,l^	T_21_	Soil + wheat straw @ 2.5% W/W Soil	1.52^c,d,e,f^
T_8_	Soil + poultry manure @ 1% W/W Soil	1.55^b,c,d,e^	T_22_	Soil + wheat straw @ 5.0% W/W Soil	1.51^d,e,f^
T_9_	Soil + poultry manure @ 2.5% W/W Soil	1.54^b,c,d,e,f^	T_23_	Soil + gypsum @ 1% W/W Soil	1.58^b^
T_10_	Soil + poultry manure @ 5.0% W/W Soil	1.51^e,f^	T_24_	Soil + gypsum @ 2.5% W/W Soil	1.56^b,c,d^
T_11_	Soil + pressmud @ 1% W/W Soil	1.53^b,c,d,e,f^	T_25_	Soil + gypsum @ 5.0% W/W Soil	1.54^b,c,d,e,f^
T_12_	Soil + pressmud @ 2.5% W/W Soil	1.51^d,e,f^	T_26_	Soil + horse waste @ 1% W/W Soil	1.32^i^
T_13_	Soil + pressmud @ 5.0% W/W Soil	1.49^f,g^	T_27_	Soil + horse waste @ 2.5% W/W Soil	1.30^i^
T_14_	Soil + biochar @ 1% W/W Soil	1.45^g,h^	T_28_	Soil + horse waste @ 5.0% W/W Soil	1.29^i^
Tukey HSD	0.048				

*FYM* Farm yard manure, *W/W* weight by weight.

The alphabets e.g., a, b, c represent the statistical grouping of treatments based on their Cd values.

### Impact of organic amendments on Ni in soil

The application of organic amendments significantly impacted soil nickel concentrations, with a consistent decreasing trend observed across treatments (Table 11). Statistical analysis confirmed significant differences among treatments. The control treatment (T1) had the highest nickel concentration of 13.52 mg kg^−1^, while the other treatments ranged from 12.87 to 10.55 mg kg^−1^. Among the tested organic amendments, treatments with farmyard manure (FYM) (T2–T4), compost (T5–T7), and horse waste (T26–T28) were the most effective in reducing nickel concentrations compared to the control. Treatments with poultry manure (T8–T10), press mud (T11–T13), rice husk (T17–T19), wheat straw (T20–T22), and gypsum (T23–T25) were less effective in reducing nickel levels, but still showed a decrease in nickel concentration compared to the control, indicating their potential for soil remediation.

**Table 11:** Impact of various treatments on Ni of soil.

Sr. No.	Treatments	Ni	Sr. No.	Treatments	Ni
T_1_	Control	13.52^a^	T_15_	Soil + biochar @ 2.5% W/W Soil	11.62^j,k^
T_2_	Soil + FYM @ 1% W/W Soil	10.63^l,m,n^	T_16_	Soil + biochar @ 5.0% W/W Soil	11.57^k^
T_3_	Soil + FYM @ 2.5% W/W Soil	10.59^n,o^	T_17_	Soil + ricehusk @ 1% W/W Soil	12.73^e,f^
T_4_	Soil + FYM @ 5.0% W/W Soil	10.55^o^	T_18_	Soil + ricehusk @ 2.5% W/W Soil	12.70^f,g^
T_5_	Soil + compost @ 1% W/W Soil	10.63^l,m,n^	T_19_	Soil + ricehusk @ 5.0% W/W Soil	12.66^g,h^
T_6_	Soil + compost @ 2.5% W/W Soil	10.62^m,n,o^	T_20_	Soil + wheat straw @ 1% W/W Soil	12.75^e,f^
T_7_	Soil + compost @ 5.0% W/W Soil	10.57^n,o^	T_21_	Soil + wheat straw @ 2.5% W/W Soil	12.72^f,g^
T_8_	Soil + poultry manure @ 1% W/W Soil	12.87^b^	T_22_	Soil + wheat straw @ 5.0% W/W Soil	12.69^f,g^
T_9_	Soil + poultry manure @ 2.5% W/W Soil	12.71^f,g^	T_23_	Soil + gypsum @ 1% W/W Soil	12.73^e,f^
T_10_	Soil + poultry manure @ 5.0% W/W Soil	12.75^d,e,f^	T_24_	Soil + gypsum @ 2.5% W/W Soil	12.59^h,i^
T_11_	Soil + pressmud @ 1% W/W Soil	12.84^b,c^	T_25_	Soil + gypsum @ 5.0% W/W Soil	12.55^i^
T_12_	Soil + pressmud @ 2.5% W/W Soil	12.82^b,c,d^	T_26_	Soil + horse waste @ 1% W/W Soil	10.70^l^
T_13_	Soil + pressmud @ 5.0% W/W Soil	12.80^c,d,e^	T_27_	Soil + horse waste @ 2.5% W/W Soil	10.69^l^
T_14_	Soil + biochar @ 1% W/W Soil	11.66^j^	T_28_	Soil + horse waste @ 5.0% W/W Soil	10.68^l,m^
Tukey HSD	0.071				

*FYM* Farm yard manure, *W/W* weight by weight.

The alphabets e.g., a, b, c represent the statistical grouping of treatments based on their Ni values.

### Impact of organic amendments on As in soil

The data revealed that all treatments had a statistically significant impact on soil arsenic concentrations (Table 12). Statistical analysis confirmed significant differences in arsenic concentrations among the various treatments. The control treatment (T1) had the highest arsenic concentration of 4.17 mg kg^−1^, while treatment T7 had the lowest concentration of 2.02 mg kg^−1^. Treatments T4 (soil + FYM at 5.0% w/w soil), T6 (soil + compost at 2.5% w/w soil), and T3 (soil + FYM at 2.5% w/w soil) also showed relatively low arsenic concentrations of 2.03, 2.08, and 2.10 mg kg^−1^, respectively. The use of horse waste was found to be second only to compost in reducing soil arsenic concentrations. However, the application of amendments such as poultry manure (T8–T10), press mud (T11–T13), rice husk (T17–T19), wheat straw (T20–T22), and gypsum (T23–T25) was less effective in reducing arsenic levels compared to farmyard manure (FYM), compost, and horse waste. Nevertheless, the arsenic concentrations in these treatments were lower than in the control, indicating their potential for soil remediation.

**Table 12:** Impact of various treatments on As.

Sr. No.	Treatments	As	Sr. No.	Treatments	As
T_1_	Control	4.17^a^	T_15_	Soil + biochar @ 2.5% W/W Soil	2.17^f,g^
T_2_	Soil + FYM @ 1% W/W Soil	2.15^f,g,h^	T_16_	Soil + biochar @ 5.0% W/W Soil	2.15^f,g,h^
T_3_	Soil + FYM @ 2.5% W/W Soil	2.10^h,i^	T_17_	Soil + ricehusk @ 1% W/W Soil	3.82^c,d^
T_4_	Soil + FYM @ 5.0% W/W Soil	2.03^j^	T_18_	Soil + ricehusk @ 2.5% W/W Soil	3.79^c,d^
T_5_	Soil + compost @ 1% W/W Soil	2.17^f,g^	T_19_	Soil + ricehusk @ 5.0% W/W Soil	3.77^d^
T_6_	Soil + compost @ 2.5% W/W Soil	2.08^i,j^	T_20_	Soil + wheat straw @ 1% W/W Soil	3.84^c^
T_7_	Soil + compost @ 5.0% W/W Soil	2.02^j^	T_21_	Soil + wheat straw @ 2.5% W/W Soil	3.82^c,d^
T_8_	Soil + poultry manure @ 1% W/W Soil	3.15^e^	T_22_	Soil + wheat straw @ 5.0% W/W Soil	3.79^c,d^
T_9_	Soil + poultry manure @ 2.5% W/W Soil	3.14^e^	T_23_	Soil + gypsum @ 1% W/W Soil	4.08^b^
T_10_	Soil + poultry manure @ 5.0% W/W Soil	3.12^e^	T_24_	Soil + gypsum @ 2.5% W/W Soil	4.06^b^
T_11_	Soil + pressmud @ 1% W/W Soil	3.12^e^	T_25_	Soil + gypsum @ 5.0% W/W Soil	4.02^b^
T_12_	Soil + pressmud @ 2.5% W/W Soil	3.12^e^	T_26_	Soil + horse waste @ 1% W/W Soil	2.19^f^
T_13_	Soil + pressmud @ 5.0% W/W Soil	3.10^e^	T_27_	Soil + horse waste @ 2.5% W/W Soil	2.16^f,g,h^
T_14_	Soil + biochar @ 1% W/W Soil	2.19^f^	T_28_	Soil + horse waste @ 5.0% W/W Soil	2.12^g,h,i^
Tukey HSD	0.062				

*FYM* Farm yard manure, *W/W* weight by weight.

The alphabets e.g., a, b, c represent the statistical grouping of treatments based on their As values.

## Discussion

It is revealed that untreated wastewater used for agricultural purposes indeed contained alarmingly high concentrations of heavy metals, presenting a severe threat to the environment and human health. These heavy metals including lead, cadmium, chromium and arsenic are well-known for their toxic effects and ability to persist in the environment. Results of current study demonstrated significant accumulation of heavy metals in the soils over time. Toxicity levels observed in both water and soil samples underscored the urgent need for effective remediation measures to reduce heavy metal contamination and mitigate its adverse effects. Beyond reducing heavy metal concentrations, current research examined the broader impact of wastewater treatment with organic amendments on soil quality. Soil health indicators such as pH, organic matter content, and nutrient levels were monitored. This improvement translated into increased crop yields, which is crucial for food security in regions where untreated wastewater is commonly used for irrigation. Some basic phenomena that revealed these positive changes in improving soil quality, enhancing its fertility and overall health with the use of wastewater irrigation are as under:

Soil reaction (pH) is the only parameter, that illuminates a complete representation of growth media for crop development counting nutrient supply tendency, the fortune of supplementary nutrients, salt position and soil airing, soil mineral composition and final climate situations of the area. Soil pH is basic in several parts of Pakistan comprised of arid and semi-arid regions. A decline in soil pH owing to any land managing approach is permanently significant and causes in eventual alteration of soil in the direction of suitable one and clear conversion into improved crops. A decrease in pH of basic soil owing to the usage of carbon-based amendments was also detected by former researchers. Smiciklas et al.^21^ explored the possibility of applying composted food, paper, landscape and manure waste to continue soil healthiness. They stated from these findings that a declining tendency in soil pH was noted in compost-treated plots as linked to control. In a similar mode, a decrease in soil pH and SAR was detected by Sarwar et al.^22^ through the usage of carbon-based materials and gypsum. They credited the reduction in soil pH and SAR of soil by carbon-based materials to the production of organic acids beginning mobilization of native calcium existing as CaCO_3_ in the soil. Pattanayak et al.^23^ also detected a decline in soil pH and subsequently the fruitage of rice crops when they appraised 3 green manure crops. They argued that the elimination of cations like Ca, Mg and organic acids formed throughout the breakdown of added green manure material had caused low pH. Likewise, Ibrahim et al.^24^ also stated a reduction in the pH of soil by the addition of green manuring and FYM. Adding carbon-based like FYM, green manure, and compost was beneficial to lessening a rise in soil pH through reactions of organic acid creation and a positive impact on soil physical parameters.

Yaduvanshi^25^ noted that inclusion of green manuring and FYM for five years abridged the soil pH on the rice–wheat cropping system. He claimed this reduction might be accredited to the advanced creation of CO_3_ and organic acids. Noted from field research that constant cropping for 03 years underneath a rice–wheat system lessened soil pH. In disparity, pH of acidic soils has been stated to be augmented subsequently adding carbon-based materials in the form of compost^26^. An upsurge in soil pH points and some macro and micro-nutrients was also observed by Irshad et al.^27^ through the usage of urban solid waste composts in the soil. Likewise, Selvakumari et al.^28^ noted that the nonstop use of fly ash caused an important rise in pH. They also stated that the extent of the rise in pH was little when carbon-based manures were combined which might be owing to the development of constant complexes of Ca and Na with CO_2_ and organic acids changed from the disintegrating carbon-based manures. The usage of carbon-based amendments in current research occasioned clear acidulating outcomes on the soil. The manufacture of organic acid during the mineralization of carbon-based materials. The pH of alkaline soil is measured by H, Fe and Al ions though that of basic soils is determined by Ca and Mg^29^ where Na is in a regulatory situation when soil is sodic too. So, free Ca will raise the Ca content in the soil solution consequential reduction of SAR. It might also precipitate as CaCO_3_ in calcareous soils. Sodium ions (free ions) may percolate deep down into the profile as all Na^+^ salts (even Na_2_CO_3_) are extremely soluble. It is completed almost for the recovery of salt-affected soils.

Electrical conductivity (EC) is a soil property that directs discursively the total contents of solvable salts and is the shortest quantity of salinity. Saltiness is an issue in arid and semi-arid regions in the world. This is a specific issue of irrigated farming in such zones. Above its critical value plants face difficulties in water uptake due to physiological unapproachability, osmotic effects due to the decline of water potential and limited nutrient uptake that might be due to specific ion effects. A tendency of overall decline in soil EC was detected in all pots as well as in field experiments. The addition of carbon-based materials principally produced a reduction of this soil property. Alike information can also be layered from the literature^28,30^, which showed that EC reduced in acidic as well as alkaline soils when carbon-based materials of diverse nature were added to the soil. The disintegration of carbon-based materials produced acids or acid-forming complexes which respond with the partially solvable salts previously existing in the soil and either change these into solvable salts or in any case upsurge their solubility and at the same time either discharge in the soil profile or absorption by plants. Nevertheless, the quantity of decline will rest on how much amount of the acids or acid-making materials was formed which will in sequence depend on the quantity of the carbon-based materials used. The other aspect to be taken into thought is the elimination of solvable salts from the soil ecosystem. If the physical characteristics of soils are good and the system is open as in the case of field experiments, the outcome of decline in soil EC will moderately be enhanced. In the case of basic soil, a net decline in the EC of soil was detected. The key factor for this reduction in soil EC that can be recommended through judgment is the escape of answerable salts into the lower profile. There is strong evidence in the works^31^ that the physical characteristics of alkaline soils significantly improve when carbon-based materials are used. The physical parameters of soil were meaningfully upgraded when carbon-based amendments were added to the soil^32^. Soil carbon-based materials boost granulation, raise cation exchange capacity, and improve soil adsorption. Cations (Ca^2+^, Mg^2+^ and K^+^ are formed throughout disintegration^27,29^.

Soil SAR is a gauge that is utilized to assess sodicity hazards. Sodicity is the buildup of sodium ions to extreme extents, which hamper plant development straight or through the damage of physical soil environments. A noticeable decline in SAR of soil was noted in all pot and field experiments. The consequence of carbon-based amendments was favorable over control. Usage of FYM and compost demonstrated superior to other materials. Studies by Sarwar et al.^33^ showed a similar tendency of lessening in soil SAR by the usage of FYM, rice straw and sesbania green manure. They accredited this decrease in SAR of the soil with carbon-based amendments due to the making of organic acids producing mobilization of natural calcium present as CaCO_3_ in the soil. Soil SAR decreases either due to a rise in divalent cations (Ca + Mg) or a decline in monovalent cations (Na). Values of cations showed that Na reduced through Ca + Mg improved after the usage of various carbon-based amendments. The chemical reactions quoted under discussion on soil pH more explain how a net rise in Ca + Mg and a decline in Na^+^ in the soil solution happened. The acid or acid-making elements ejected Na^+^ or Ca^+2^ + Mg^+2^ from the clay micelle, the hydrogen ion taking their place. Sodium salts being willingly water soluble left the soil system and went into the lower depths of the soil profile. The divalent cations (Ca + Mg) amplified the net contents in the soil solution. Nevertheless, a portion of these would have also been triggered by carbonates and bicarbonates existing in the soil^33^.

All above improvements were noted as the result of addition of various organic amendments like FYM, compost, biochar, horse waste, poultry manure etc. Compost is a stable humic substance which helps in accelerating soil microbial and enzyme activities. It is a commonly used soil amendment in poor urban areas due to its low cost and convenience. Compost is known to increase soil organic matter and improve soil structure; thus, there is enhanced plant growth. In addition, soil amendments such as biochar, compost and their mixture can also immobilize heavy metals. Although there are several studies on the role of biochar and compost in immobilizing heavy metal, Still, little work has been done mainly on Pakistani soil to compare the sole effect of both organic amendments (biochar and compost) on the immobilization of heavy metals like Pb, Cd, and Cr in soil and their toxicity to plants. Research was done to explore efficiency of organic amendments (biochar and compost) for their effect on Pb, Cd, and Cr immobilization in soil, photosynthesis, and maize growth^34^.

An organic amendment (biochar), a carbonaceous material made by pyrolyzing organic waste materials, is being promoted as a soil conditioner to enhance soil fertility, water holding capacity, nutrient retention, and soil C storage^35^. In addition, biochar has also been shown to decrease the toxicity of heavy metals in soil^36^. Since adsorption capacity of biochar is much higher than its precursors, the leachability and bioavailability of heavy metals can be reduced because biochar has large surface area to adsorb heavy metals and organic pollutants. It can, therefore, be imagined that the biochar materials would also be capable of adsorbing metals derived from metal-oxide nanoparticles thereby reducing their toxicity^37^ and^38^). It is concluded that organic amendments (biochar and compost) decreased heavy metals availability in the soil, reducing toxicity in the plant. However, biochar was most effective in reducing heavy metals content in soil and plant compared to compost^34^.

The data revealed that the uptake of major nutrients such as nitrogen, phosphorus, and potassium was enhanced, as their concentrations were significantly higher compared to the control treatment. The addition of farmyard manure (FYM) and compost proved to be the most effective among all amendments, as evident from plant analysis, which showed superior nutrient uptake compared to other treatments^39^. Soil pH played a crucial and positive role in enhancing nutrient uptake by plant roots, as the soil had already been adjusted to a neutral range from alkaline, making nutrients more readily available for plants. This favorable change in soil pH increased the solubility of various nutrients, particularly phosphorus. The decrease in soil pH led to the conversion of soil phosphorus from PO_4_^3−^ to HPO_4_^2−^ or even H_2_PO^4−^ for a brief period, resulting in higher phosphorus uptake by plants. Moreover, the decomposition of organic amendments released more H ions, leading to the release of potassium from fixed positions or exchange sites of soil clay. Notably, several nutrients become more readily available when soil pH is near the neutral to acidic range^40^. During the process of disintegration of organic amendments, some organic acids are released which make the rhizosphere pH in acidic range and all nutrients are converted into available form and readily taken up by plants. The presence and release of organic acids upgrade all soil physical properties that too contribute to enhanced plant growth through more root activities. Further, K^+^ plays its role in the regulatory process of closing and opening of stomata which eventually results in more concentration and uptake of other nutrients^41^. Organic amendments incorporation in contaminated soil benefits over inorganic due to cost-effectiveness, higher biodegradability, and enhancement in soil properties. It has been reported that the accumulation of toxic metals was significantly decreased, and maize growth, biomass production increased with biochar application. Different organic amendments like biochar and compost have also been reported as potential soil amendments for reducing bioavailable fractions of Pb, Zn, and Cd in the soil^34^.Recent studies have investigated the use of chemical additives and organic fertilizers to mitigate heavy metal pollution in soil and its impact on crop growth and antioxidant activity. Hong et al.^42^ explored the effects of chemical additives on soil heavy metal pollution and lettuce antioxidant activity. Taeprayoon et al.^43^ examined the use of organic fertilizers in cadmium-contaminated soils for phytoremediation of bioenergy crops, while^44^ tested the effectiveness of organic additives in stabilizing cadmium concentrations and promoting bioenergy crop growth in contaminated soils. Wang et al.^45^ studied the impact of soil fertilizers on heavy metal fixation and accumulation in maize grown in multi-metal-contaminated soils. Van Zwieten^46^ emphasized the importance of organic materials in addressing soil problems for sustainable crop production. This study highlights the crucial role of organic fertilizers in enhancing soil fertility and ensuring sustainable crop productivity.

## Conclusions

Based on findings it has been recommended that farmers can use wastewater after treatment with organic amendments (FYM and compost) as compared to inorganic ones in a better way. The soil health and quality can be improved and maintained through the application of organic amendments (used in this study) as compared to inorganic ones in a better way. Among the organic amendments, application of FYM and compost proved more effective regarding soil health as its addition reduced the concentration of heavy metals (lead, cadmium, nickel and arsenic) in soil samples.

## Data Availability

All data generated or analyzed during this study are included in this published article.
