# Author Correction: Efficacy of various amendments for immobilization of potentially toxic elements in wastewater contaminated soils

**DOI:** 10.1038/s41598-024-71814-4

**Published:** 2024-09-05

**Authors:** Muhammad Zeeshan Manzoor, Ghulam Sarwar, Salman Alamery, Muhammad Ibrahim, Amtul Sami, Bilal Ahmed, Fariha Ahsan, Salma Gul, Kotb A. Attia, Sajid Fiaz, Ikram Ullah

**Affiliations:** 1 Department of Soil and Environmental Sciences, College of Agriculture, University of Sargodha, Sargodha, Pakistan; 2 Department of Biochemistry, College of Science, King Saud University, P.O. Box 2455, 11451 Riyadh, Saudi Arabia; 3 Department of Environmental Sciences, Government College University Faisalabad, Faisalabad, Pakistan; 4 Department of Health Biotechnology, Women University Swabi, Swabi, Pakistan; 5 Department of Structural Engineering, Faculty of Civil Engineering, Doctoral School, Silesian University of Technology, Akademicka 2, 44‑100 Gliwice, Poland; 6 Institute of Microbiology, University of Sindh, Jamshoro, 76080 Pakistan; 7 National Centre of Excellence in Physical Chemistry, University of Peshawar, Peshawar, Pakistan; 8 Department of Plant Breeding and Genetics, The University of Haripur, Haripur, 22620 Pakistan; 9 Department of Landscape and Horticulture, Yunnan Agricultural University, Kunming, 650201 China

Correction to: *Scientific Reports* 10.1038/s41598-024-65686-x, published online 29 July 2024

In the original version of this Article Kotb A. Attia was incorrectly affiliated with ‘Department of Environmental Sciences, Government College University Faisalabad, Faisalabad, Pakistan’. The correct affiliation is listed below.

Department of Biochemistry, College of Science, King Saud University, P.O. Box 2455, 11451 Riyadh, Saudi Arabia.

The original Article has been corrected.

